# The challenge of chronic lung disease in HIV-infected children and adolescents

**DOI:** 10.7448/IAS.16.1.18633

**Published:** 2013-06-18

**Authors:** Heinrich C Weber, Robert P Gie, Mark F Cotton

**Affiliations:** 1 Rural Clinical School, School of Medicine, Faculty of Health Sciences, University of Tasmania, Burnie, Tasmania, Australia; 2 Department of Paediatrics and Child Health, Faculty of Medicine and Health Sciences, Stellenbosch University, Tygerberg Children's Hospital, Tygerberg, South Africa

**Keywords:** HIV, adolescent, children, chronic lung disease, LIP, bronchiectasis, tuberculosis (TB)

## Abstract

Until recently, little attention has been given to chronic lung disease (CLD) in HIV-infected children. As the HIV epidemic matures in sub-Saharan Africa, adolescents who acquired HIV by vertical transmission are presenting to health services with chronic diseases. The most common is CLD, which is often debilitating.

This review summarizes the limited data available on the epidemiology, pathophysiology, clinical picture, special investigations and management of CLD in HIV-infected adolescents. A number of associated conditions: lymphocytic interstitial pneumonitis, tuberculosis and bronchiectasis are well described. Other pathologies such as HIV-associated bronchiolitis obliterans resulting in non-reversible airway obstruction, has only recently been described.

In this field, there are many areas of uncertainty needing urgent research. These areas include the definition of CLD, pathophysiological mechanisms and common pathologies responsible. Very limited data are available to formulate an effective plan of investigation and management.

## Introduction

As the HIV epidemic matures in sub-Saharan Africa, adolescents with undiagnosed HIV disease may present to health services. The majority of these HIV-infected youth have severe immunosuppression and a heavy burden of chronic complications, with the most common chronic complications being growth failure, lung and cardiac disease [1]. It is postulated that this represents an emerging, previously unrecognized severe burden of disease in countries with a high HIV burden. It is estimated that 36% of perinatally infected children may be slow progressors, who would have a mean survival of 16 years without combination of antiretroviral therapy (ART) [2]. From using data collected in Zimbabwe and South Africa, modelling suggests that a substantial number of previously undiagnosed HIV-infected adolescents will present with chronic disease, with a high percentage having chronic lung disease (CLD) [2]. This problem is likely to increase over time, as currently only 30% of children requiring ART are treated in sub-Saharan Africa [3] and with increasing ART use, more children will reach adolescence and will contribute to the pool of adolescents with chronic diseases including CLD.

In an excellent review of non-infectious CLD in HIV-infected children, lymphocytic interstitial pneumonitis (LIP), malignancies, immune reconstitution inflammatory syndrome (IRIS), bronchiectasis, interstitial pneumonitis and aspiration pneumonia were described [4]. That review clearly describes the available information on the pathogenesis, clinical picture, and management of these conditions and will not be the focus of this review. The aim of this review is to highlight the magnitude of the CLD in HIV-infected children and adolescents, review the diseases that contribute to the spectrum of CLD, their possible pathogenesis, clinical features and management.

## Methods

Electronic search was done using the keywords: HIV-infected, children, adolescents, CLD, LIP, bronchiectasis and tuberculosis (TB). Only articles in English and those electronically available were selected. The search engines used were PubMed and Google Scholar.

## Epidemiology of CLD in children and adolescents

Very few studies have extensively examined the epidemiology of CLD in children and adolescents. In a study of 301 adolescents (10–18 years) hospitalized in Zimbabwe, 41% were HIV-infected. In view of a high prevalence of stunting and either being orphaned or having an HIV-infected mother, perinatal HIV acquisition was felt to be most likely in 80% of these infected youth [5]. Of 116 consecutive adolescents attending two outpatient clinics in Harare investigated for CLD, 71% were between 13 and 18 years of age, all were stunted and 69% were on ART [6]. The vast majority, 86%, met study definition for CLD. Similar data are now emerging from Malawi where, in a study of 79 consecutive adolescents, over 50% reported dyspnea sufficient to limit their daily activities and a third had abnormal lung function tests [7]. No gender differences have been noted for CLD disease in HIV-infected children and adolescents [6–14].

Due to the limited data available, the full impact of HIV-related CLD cannot at present be estimated. However, from emerging data it seems likely that adolescent survivors of perinatal HIV infection an increasing prevalence of chronic disease, of which CLD is the largest burden. Health services are unlikely to be geared up to diagnose and manage this emerging epidemic of CLD amongst HIV-infected adolescents.

## Defining CLD

**Figure 1:**
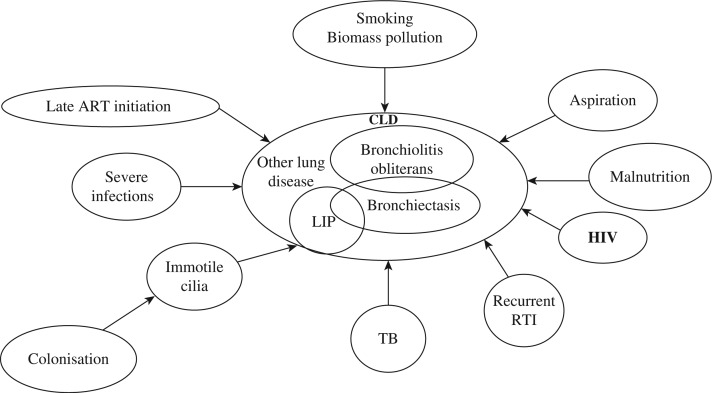
Potential pathophysiologic mechanisms leading to CLD in HIV-infected children and adolescents. **RTI, recurrent respiratory tract infection**.

CLD is a non-specific term that does not define the underlying pathology, but only suggests that there is an underlying chronic lung condition present. The symptoms associated with CLD such as cough or breathlessness could equally be due to chronic cardiac disease. Radiological criteria to define CLD are also problematic as they are observer dependent and terminology in radiological abnormalities varies between readers. Changes associated with bronchiectasis, LIP and bronchiolitis are not precise and subject to interpretation. Many factors contributing to CLD are shown in Figure 1.

Therefore, in this review we suggest that the definitions used by Ferrand et al., who have published the most extensive article on CLD in adolescents, should be used as a screening tool [6]. Suspected CLD should include two or more of the following features:

1. Chronic cough (defined as a cough present most days for three months per year in the past two years);
2. Recurrent respiratory tract infections (>two antibiotic courses in the last year);
3. Moderate to severe limitation in physical activity caused by breathlessness (New York Heart Association class 2–4);
4. Existing diagnosis and/or signs of cor pulmonale;
5. Hypoxia (O_2_ saturation ≤92%) at rest or desaturation (O_2_ saturation ≥5% reduction) on exercise.

The limitation of this definition is that data on all the elements are not available in primary care clinics in many parts of the world where HIV prevalence is highest. Children with the clinical elements of CLD should be referred to regional/tertiary hospitals to confirm CLD, determine its cause and exclude other possible causes such as chronic cardiac disease.

## Conditions associated with CLD

### Lymphoid interstitial pneumonitis

LIP was described in the 1980s in children with (AIDS), predating diagnostic tests for HIV. The first descriptions indicate that children with LIP were older than those presenting with *Pneumocystis jerovicii* pneumonia (PCP) and had a better outcome, as LIP occurred in slow progressors and was associated with a relatively preserved CD4 cell count [8]. LIP is thought to be a lymphoproliferative response to HIV or Epstein-Barr virus (EBV) [15]. Children with LIP had chronic cough, tachypnoea, clubbing and accompanying hypoxia. As part of a lymphoproliferative response, an interstitial pneumonitis, generalised lymphadenopathy, parotid swelling and hepatosplenomegaly were also noted.

The typical chest x-ray (CXR) and CT scan appearance is of a diffuse, symmetrical reticulonodular or nodular pattern. Bronchiectasis is a recognised complication in children in 12.5% of cases [8]. The pathogenesis of the bronchiectasis remains unclear. As children with LIP have repeated lower respiratory tract infections, it is uncertain if it is the repeated infections that result in bronchiectasis or HIV itself. LIP responds well to ART [16].

### Bronchiectasis

Bronchiectasis is characterized by permanent and abnormal widening of bronchi due to loss of elastin and more advance disease by destruction of muscle and cartilage [17]. The first descriptions in HIV-infected children were from two retrospective case series in the United States (USA). Between 1981 and 1990, 32 out of 77 children had LIP in the first series. Four children with LIP had bronchiectasis confirmed by CT scan [8]. The second study, conducted in 1997, of HIV-infected children referred to a pulmonology clinic noted bronchiectasis in 26 (15.8%) out of 164 children. Common predisposing factors were LIP and recurrent and unresolved pneumonia [14]. In a more recent retrospective study, bronchiectasis occurred in 5.7% of children. Mean age at first presentation with HIV-disease was 2.1 years, with age 7.8 years when bronchiectasis was first diagnosed [9]. The risk factors for developing bronchiectasis were recurrent pneumonia, severe immunosuppression and LIP [9].

Bronchiectasis was not thought to be common in Africa. Cases were first described by Kiwanuka et al. who reported bronchiectasis in five out of 110 children investigated for TB [11]. Recently, data was published on 35 HIV-infected children with bronchiectasis. Median age was 6.9 years; all were already on ART, but continued to have significant morbidity [12]. The aetiology of the bronchiectasis was not explored.

### TB as a cause and complicating disease of CLD

In adult studies, lung function impairments were noted following TB treatment, with increasing impairment occurring with recurrent episodes of TB [18]. Ehrlich et al. noted a combined obstructive/restrictive lung function pattern in adults after TB [19]. Whether this is also true for adolescent patients is uncertain.

TB was commonly implicated as a cause of bronchiectasis preceding the HIV pandemic [12, 20]. In HIV-infected children with symptoms and signs of CLD not responding initially to three months of “standard” treatment including for TB, TB was still confirmed in 29% by lung biopsy [10]. Similarly, in a case series of older HIV-infected children receiving ART complicated by bronchiectasis, 36% were previously treated for TB, with microbiological confirmation in 11% of cases. These two studies highlight the difficulty in making and confirming the diagnosis of TB in HIV-infected children with CLD [12]. The relationship between pulmonary TB and bronchiectasis remains unclear in adolescent HIV-infected patients, but a history of treatment for TB is common in such patients [6].

Regardless of the role of TB in CLD pathogenesis, the diagnosis of TB should always be considered and actively evaluated. TB can cause bronchiectasis through airways destruction and patients with HIV-related CLD can acquire TB at any time.

### Bronchiolitis obliterans

Bronchiolitis obliterans is characterised by fibrotic constriction and/or complete obstruction of the bronchioles [21]. In children, it is usually linked to events in the first two years of life, especially severe adenoviral and mycoplasma pneumonia requiring supplemental oxygen and assisted ventilation [21, 22]. These children have small airway disease with significant air trapping and wheezing. Co-existing bronchiectasis occurs commonly. The ongoing clinical course of post-infective bronchiolitis obliterans in HIV-infants and children still needs to be described. In the Zimbabwean study of HIV-infected adolescent patients with CLD, bronchiolitis obliterans was the most common cause of CLD, although many adolescents had co-existing radiological features of bronchiectasis. The authors speculate that the bronchiolitis obliterans was caused by multiple bacterial and/or viral infections. HIV immunosuppression might also contribute by facilitating ongoing small airway inflammation. A further hypothesis is that a progressive inflammatory bronchiolitis obliterans may be caused by HIV itself. Further research to delineate the cause and effective management of bronchiolitis obliterans in HIV-related CLD is needed [6].

### Chronic aspiration pneumonia

Children with HIV are also at increased risk for esophagitis due to *Candida albicans* and cytomegalovirus. In a radiological review, aspiration pneumonia was proposed as cause of CLD frequently associated with gastro-oesophageal reflux disease in HIV-infected children [23, 24]. In a case series describing swallowing problems in 25 HIV-infected young children, 83% (15/18) of children referred for assessment due to recurrent respiratory tract infections had swallowing dysfunction [25].

### Interstitial lung disease

Other interstitial lung diseases rarely occur in HIV-infected children and adolescents. Their clinical picture would be difficult to distinguish form other causes of interstitial lung disease or infection [4]. For an accurate diagnosis, an open lung biopsy is required, but rarely performed in HIV-infected children.

### Special investigations

#### Radiology

In a prospective study of HIV-infected children in the United States from the pre-ART era, a progressive accumulation of chronic CXR abnormalities, defined as abnormalities lasting more than three months, was present in children by four years of age in 32.8% of 86 HIV-infected children followed from birth [13]. The most common abnormalities were increased bronchovascular markings and reticular densities. Risk factors for chronic CXR abnormalities were declining CD4 count and increasing viral load [13].

Ferrand et al. found radiological abnormalities in 51 (68%) out of 75 adolescents, of which 74% were classified as severe. The most common reported abnormalities were ring/tramline opacities and alveolar consolidation. Predominant consolidation was associated with progressive dyspnea (odds ratio 5.6 (95% CI 1.6–20)) [26]. In a subsequent study, using high-resolution computer tomography (HRCT) in adolescents with suspected CLD, the major finding was decreased attenuation, consistent with small airway disease, most likely representing obliterative bronchiolitis [6]. The next most common findings were consistent with large airway abnormalities, i.e., bronchial wall thickening, small and large airway plugging, indicating bronchiectasis (43%).

More specific radiological features, associated with specific diagnoses such as LIP, bronchiectasis, TB, interstitial lung disease and malignancies are commonly present in these patients. The interpretation of CXR findings in HIV-infected children is complicated by persistent radiological changes and an increased range of chronic respiratory tract pathologies including TB and acute lower respiratory tract infections. In a recent review, the authors appealed for greater cooperation between clinicians and radiologists to facilitate interpretation of the chest radiographic findings and to avoid unnecessary mistakes [27].

#### Lung function testing

In the earliest report of lung function in HIV-infected children, the airway resistance in the HIV-infected children was higher when compared to that in HIV-uninfected children (0.84±0.3 vs. 0.64±0.08 kPa L^−1^s; *p*<0.0001) [28]. The airway resistance declined in HIV-uninfected patients over time, but increased in the HIV-infected children, suggesting ongoing pathology. The extent of airway resistance was associated with the duration of HIV infection, rather than intercurrent infections, suggesting that HIV itself might contribute to ongoing airway disease.

Evidence of large airway obstruction has also been noted. In the Zimbabwean study of HIV-infected adolescents, 33% had a peak expiratory flow rate (PEFR) below 80% of predicted, and 45% had forced expiratory volume in 1 second (FEV_1_) below 80% of predicted [6]. In a similar study of 79 HIV-infected youth in Malawi (median age 10.8 years), abnormal spirometry was detected in 33% [7]. Of these, almost a third (31%) had non-reversible airway obstruction as demonstrated by no bronchodilator response and suggesting structural lung disease in this group. In this study, 22% had hypoxia at rest and a further 35% desaturated on walking [7]. In a study of HIV-infected children with bronchiectasis in South Africa, the median FEV_1_% was only 53% of predicted (range: 5–86%) and median mid-expiratory flow rate (FEF_25–75%_) was 52% (range 11–165%) [12]. This study suggests both large and small structural airways disease.

The measurement of oxygen saturations, before and after exercise, should be included in evaluating patients suspected of CLD, as at least one third might be missed if symptom-based screening is used [7].

There are no follow-up lung function studies of children or adolescents with CLD to investigate the progression of lung disease, especially in older children and adolescents receiving ART. These studies are important to follow the course of structural lung disease and investigate if early ART would prevent CLD, especially bronchiolitis obliterans, developing. A paucity of lung function data limits the predictions of the progression of disease and the response to treatment.

#### Microbiology

In investigating the organisms isolated from sputum in HIV-infected children with bronchiectasis in South Africa, *Haemophilus influenzae* and *H*. *parainfluenzae* accounted for 49% of the isolates while *Pseudomonas aeruginosa* (2%), *Staphylococcus aureus* (2%) and *Mycobacterium tuberculosis* (1%) were rarely isolated [12]. In a study of HIV-infected adolescents in Zimbabwe, sputum cultures were positive for bacteria or fungi in 18 (46%) and mycobacteria in 12 (22%) of 54 samples [6]. *H. influenzae, Moraxella and S. aureus* were the most common bacterial isolates. *Mycobacterium tuberculosis* was found in eight adolescents, of whom seven were also sputum Ziehl-Neelsen positive. In addition, the sputum was smear positive in 16% of cases from whom no Mycobacterial species was cultured. These adolescents were treated for TB [6]. This study indicates that there might be a large burden of mycobacterial disease other than TB disease in adolescents with CLD, requiring further investigation.

*Haemophilus influenza* has been implicated as a ciliotoxic bacterium, causing secondary immotile ciliary dysfunction [17]. The role of chronic bronchial infection with this and other organisms in the progression of CLD needs to be investigated as this could have implications in the treatment of CLD in HIV-infected adolescents.

#### Echocardiography

Echocardiographic evidence of pulmonary hypertension has been documented in 7% of adolescents with CLD [6]. In a study on the echocardiographic study findings in consecutive HIV-infected Zimbabwean adolescents, abnormal left ventricular (LV) hypertrophy (67%), impaired LV relaxation or restrictive LV physiology, and right ventricular dilatation without pulmonary artery hypertension (29%) were reported [29]. This data partially explains why limited ability to exercise, breathlessness and desaturation on exercise are common in HIV-infected adolescents even with minimal changes on the chest radiograph. Of note, CLD was not reported in this study. There are no reports of obstructive sleep apnea in HIV-infected adolescents that might complicate their CLD. Adult reports suggest that this may occur [30], but studies in children and adolescents are required.

### The role of ART

The Children with HIV Early Antiretroviral (CHER) trial, which commenced in 2005 and ended in 2011, emphasized the importance of early diagnosis and early ART initiation by seven weeks of age, which reduced the risk of death or disease progression by 76% compared to a deferred strategy with initiation of ART at a median of six months of age. The incidence of TB was 50% lower in infants on early ART than when deferred [31]. Benefits of early ART were sustained. After a median follow-up of 4.8 years, there were five cases of CLD, including two with bronchiectasis and one with LIP in 377 trial participants [32]. It is likely that early ART is important in protecting the lungs against damage from frequent intercurrent and severe infections.

Data from CLD studies strongly suggest that delayed initiation of ART in older children and adolescents will not improve lung function in the short-term, even with excellent adherence [6, 12].

Paradoxically, ART has been linked to deteriorating lung disease. In IRIS, pulmonary infiltrates will temporarily increase, as has been described for pulmonary TB [33, 34]. In a cross-sectional study of adults with airways obstruction, ART was associated with increased airways obstruction [35]. These data, however, require prospective studies for confirmation.

### Challenges and limitations

#### Methodological issues

The major limitation of all the cross-sectional studies is that temporality cannot be determined. Also, many studies are uncontrolled. A selection bias is likely as many studies focus on patients with severe disease. Therefore, detailed prospective studies are required.

#### Definitions of CLD

At present there are no agreed definitions of CLD in HIV-infected adolescents. The definitions would require high sensitivity and be applicable in primary care clinics in rural settings. Identified children and adolescents would require referral to regional/tertiary institutions for further investigations. These should include chest radiography, saturation monitoring (at rest, during exercise and during sleep), sputum culture for bacteria and mycobacteria, lung function testing and electrocardiography (EKG). In selected cases HRCT and echocardiography will be required. An algorithm to investigate children and adolescents should be developed and scientifically tested to ensure high sensitivity and specificity.

#### Response to therapy

There are no randomized controlled studies available to guide therapy. Although it seems logical to follow the guidelines for management of non-cystic fibrosis bronchiectasis, there is no evidence to support this approach. The postulated ongoing airways inflammation in bronchiolitis obliterans might respond to immunomodulation with macrolide antibiotics and should be studied [36]. Early identification of HIV-infected infants and children and immediate initiation of ART regardless of CD4 count may be essential to prevent CLD. Justification for this approach is the lack of association between the CD4 count, duration of ART and lung function tests indicating irreversible lung damage in adolescent patients with CLD [6].

#### Pathogenesis

A number of risk factors that commonly occur in HIV-infected children and adolescents could lead to CLD. These include repeated respiratory tract infections, LIP, pulmonary TB, poor nutrition and exposure to increased biomass pollution at home. The risk of developing CLD would be exacerbated by tobacco smoke, late initiation of ART and late recognition of the symptoms and signs suggestive of CLD. Urgent research is required to elucidate which factors are responsible for the development of CLD in adolescence and which interventions will prevent the progression of CLD.

### Management

To initiate the correct therapy, adolescents and children with symptoms and signs suggestive of CLD should be carefully evaluated and the most appropriate treatment initiated. This requires that patients with suspected CLD be referred to regional/tertiary hospitals for evaluation and treatment. In many parts of sub-Saharan Africa, facilities to make an accurate diagnosis are lacking. Therefore, at present it seems logical to use guidelines recommended for management of children with non-cystic bronchiectasis [37].

The aims of the treatment as modified from the above guidelines should be:

1. To identify and treat underlying cause to prevent disease progression;
2. To maintain or improve lung function;
3. To reduce exacerbations and improve quality of life by reducing daily symptoms;
4. To ensure optimal ART and preventative treatment including trimethoprim-sulphamethoxazole [38, 39] and isoniazid preventative treatment [40].

Briefly, this would include patient education, aggressive treatment of acute bacterial exacerbations and annual immunization against influenza and pneumococcal vaccination every five years. Home-based physiotherapy and airway clearance techniques, bronchodilators for those with reversible airway obstruction and long-term home oxygen therapy, where possible, should be prioritized. Acute bacterial exacerbations of lower airway infections should be treated with antibiotics for 14 days. The choice of antibiotics should be guided by sputum cultures.

Children and adolescents should be hospitalized if the work of breathing has significantly increased, if supplementary oxygen is required, if unable to take oral antibiotics or requires intravenous antibiotics or if a complication has developed. The most common complications are respiratory failure, haemoptysis and pulmonary hypertension leading to cor pulmonale. Protocols to manage these complications, appropriate to the level of care needed should be developed. Patients requiring long-term oral antibiotics, nebulised antibiotics, inhaled corticosteroids or possible lung resection of localized disease should be referred to tertiary institutions for advice.

All of the above-suggested treatment strategies require scientific validation, preferably in randomized control trials. Prior to this occurring, databases similar to those used in national cystic fibrosis registers might be helpful in identifying timely interventions that will benefit adolescents with CLD.

## Conclusions

CLD in HIV-infected children and adolescents requires heightened awareness to clinically identify children and adolescent patients at an early stage to prevent ongoing lung function loss. Sub-clinical CLD is likely to be common and markers of early lung damage require exploration. The success of this new era in the management of HIV-infected children and adolescents will be determined by our efforts to improve quality of life, with CLD requiring urgent attention.

## References

[1] Ferrand RA, Luethy R, Bwakura F, Mujuru H, Miller RF, Corbett EL (2007). HIV infection presenting in older children and adolescents: a case series from Harare, Zimbabwe. Clin Infect Dis.

[2] Ferrand RA, Corbett EL, Wood R, Hargrove J, Ndhlovu CE, Cowan FM (2009). AIDS among older children and adolescents in Southern Africa: projecting the time course and magnitude of the epidemic. AIDS.

[3] UNAIDS: UNAIDS report on the global AIDS epIdemIc: 2012 (2012).

[4] Zar HJ (2008). Chronic lung disease in human immunodeficiency virus (HIV) infected children. Pediatr Pulmonol.

[5] Ferrand RA, Bandason T, Musvaire P, Larke N, Nathoo K, Mujuru H (2010). Causes of acute hospitalization in adolescence: burden and spectrum of HIV-related morbidity in a country with an early-onset and severe HIV epidemic: a prospective survey. PLoS Med.

[6] Ferrand RA, Desai SR, Hopkins C, Elston CM, Copley SJ, Nathoo K (2012). Chronic lung disease in adolescents with delayed diagnosis of vertically acquired HIV infection. Clin Infect Dis.

[7] Rylance J, Mwalukomo T, Rylance S, Matchere P, Thindwa D, Webb E (2012). Lung Function and Bronchodilator Response in Perinatally HIV-infected Adolescents: Malawi. Programs and abstracts of the 19th Conference on Retroviruses and Opportunistic Infections (CROI 2012).

[8] Amorosa JK, Miller RW, Laraya-Cuasay L, Gaur S, Marone R, Nosher JL, L.D. F (1992). Bronchiectasis in children with lymphocytic interstitial pneumonia and acquired immunodeficiency syndrome. Pediatr Radiol.

[9] Berman DM, Mafut D, Djokic B, Scott G, Mitchell C (2007). Risk factors for the development of bronchiectasis in HIV-infected children. Pediatr Pulmonol.

[10] Jeena PM, Coovadia HM, Thula SA, Blythe D, Buckels NJ, Chetty R (1998). Persistent and chronic lung disease in HIV-1 infected and uninfected African children. AIDS.

[11] Kiwanuka J, Graham SM, Coulter JB, Gondwe JS, Chilewani N, Carty H (2001). Diagnosis of pulmonary tuberculosis in children in an HIV-endemic area, Malawi. Ann Trop Paediatr.

[12] Masekela R, Anderson R, Moodley T, Kitchin OP, Risenga SM, Becker PJ (2012). HIV-related bronchiectasis in children: an emerging spectre in high tuberculosis burden areas. Int J Tuberc Lung Dis.

[13] Norton KI, Kattan M, Rao JS, Cleveland R, Trautwein L, Mellins RB (2001). Chronic radiographic lung changes in children with vertically transmitted HIV-1 infection. AJR Am J Roentgenol.

[14] Sheikh S, Madiraju K, Steiner P, Rao M (1997). Bronchiectasis in pediatric AIDS. Chest.

[15] Andiman WA, Eastman R, Martin K, Katz BZ, Rubinstein A, Pitt J (1985). Opportunistic lymphoproliferations associated with Epstein-Barr viral DNA in infants and children with AIDS. Lancet.

[16] Dufour V, Wislez M, Bergot E, Mayaud C, Cadranel J (2003). Improvement of symptomatic human immunodeficiency virus-related lymphoid interstitial pneumonia in patients receiving highly active antiretroviral therapy. Clin Infect Dis.

[17] King PT (2009). The pathophysiology of bronchiectasis. Int J Chron Obstruct Pulmon Dis.

[18] Hnizdo E, Singh T, Churchyard G (2000). Chronic pulmonary function impairment caused by initial and recurrent pulmonary tuberculosis following treatment. Thorax.

[19] Ehrlich RI, Adams S, Baatjies R, Jeebhay MF (2011). Chronic airflow obstruction and respiratory symptoms following tuberculosis: a review of South African studies. Int J Tuberc Lung Dis.

[20] Dickey LB (1952). Primary pulmonary tuberculosis as a cause of bronchiectasis in children. Dis Chest.

[21] Moonnumakal SP, Fan LL (2008). Bronchiolitis obliterans in children. Curr Opin Pediatr.

[22] Fischer GB, Sarria EE, Mattiello R, Mocelin HT, Castro-Rodriguez JA (2010). Post infectious bronchiolitis obliterans in children. Paediatr Respir Rev.

[23] Pitcher RD, Goddard E, Hendricks M, Lawrenson J (2009). Chest radiographic pulmonary changes reflecting extrapulmonary involvement in paediatric HIV disease. Pediatr Radiol.

[24] Theron S, Andronikou S, George R, du Plessis J, Goussard P, Hayes M (2009). Non-infective pulmonary disease in HIV-positive children. Pediatr Radiol.

[25] Nel ED, Ellis A (2012). Swallowing abnormalities in HIV infected children: an important cause of morbidity. BMC Pediatr.

[26] Desai SR, Copley SJ, Barker RD, Elston CM, Miller RF, Wells AU (2011). Chest radiography patterns in 75 adolescents with vertically-acquired human immunodeficiency virus (HIV) infection. Clin Radiol.

[27] Dramowski A, Morsheimer MM, Frigati L, Schaaf HS, Rabie H, Sorour G (2009). Radiology services for children in HIV- and TB-endemic regions: scope for greater collaboration between radiologists and clinicians caring for children. Pediatr Radiol.

[28] de Martino M, Veneruso G, Gabiano C, Frongia G, Tulisso S, Lombardi E (1997). Airway resistance and spirometry in children with perinatally acquired human immunodeficiency virus-type 1 infection. Pediatr Pulmonol.

[29] Miller RF, Kaski JP, Hakim J, Matenga J, Nathoo K, Munyati S (2013). Cardiac disease in adolescents with delayed diagnosis of vertically acquired HIV infection. Clin Infect Dis.

[30] Epstein LJ, Strollo PJ, Donegan RB, Hendrix C, Westbrook RB (1995). Obstructive sleep apnea in patients with human immunodeficiency virus (HIV) disease. Sleep.

[31] Violari A, Cotton MF, Gibb DM, Babiker AG, Steyn J, Madhi SA (2008). Early antiretroviral therapy and mortality among HIV-infected infants. N Engl J Med.

[32] Cotton M, Violari A, Gibb D, Otwombe K, Josipovic D, Panchia R (2012). Early ART followed by Interruption Is Safe and Is Associated with Better Outcomes than Deferred ART in HIV+ Infants: Final Results from the 6- Year Randomized CHER Trial, South Africa. Programs and abstracts of the 19th Conference on Retroviruses and Opportunistic Infections (CROI 2012).

[33] Lawn SD, Bekker LG, Miller RF (2005). Immune reconstitution disease associated with mycobacterial infections in HIV-infected individuals receiving antiretrovirals. Lancet Infect Dis.

[34] Zampoli M, Kilborn T, Eley B (2007). Tuberculosis during early antiretroviral-induced immune reconstitution in HIV-infected children. Int J Tuberc Lung Dis.

[35] George MP, Kannass M, Huang L, Sciurba FC, Morris A (2009). Respiratory symptoms and airway obstruction in HIV-infected subjects in the HAART era. PLoS One.

[36] Healy DP (2007). Macrolide immunomodulation of chronic respiratory diseases. Curr Infect Dis Rep.

[37] Hill AT, Pasteur M, Cornford C, Welham S, Bilton D (2011). Primary care summary of the British Thoracic Society Guideline on the management of non-cystic fibrosis bronchiectasis. Prim Care Respir J.

[38] Chintu C, Bhat GJ, Walker AS, Mulenga V, Sinyinza F, Lishimpi K (2004). Co-trimoxazole as prophylaxis against opportunistic infections in HIV-infected Zambian children (CHAP): a double-blind randomised placebo-controlled trial. Lancet.

[39] Bwakura-Dangarembizi M, Kendall L, Bakeera-Kitaka S, Nahirya-Ntege P, Keishanyu R, Kekitiinwa A (2013). Randomized comparison of stopping vs continuing cotrimoxazole prophylaxis among 758 HIV+ children on long-term ART: the anti-retroviral research for Watoto trial. Programs and abstracts of the 20th Conference on Retroviruses and Opportunistic Infections (CROI 2013).

[40] World Health Organization (2011). Guidelines for intensified tuberculosis case-finding and isoniazid preventive therapy for people living with HIV in resource-constrained settings.

